# More on Current Evidences on Probiotics as a Novel Treatment for Non-Alcoholic Fatty Liver Disease

**DOI:** 10.5812/hepatmon.13780

**Published:** 2013-08-27

**Authors:** Pietro Vajro, Claudia Mandato, Roberta D’Aniello

**Affiliations:** 1 Department of Medicine and Surgery, University of Salerno, Baronissi-Salerno, Italy; 2 AORN Santobono Pediatric Hospital, Naples, Italy

**Keywords:** Probiotics, Non-Alcoholic Fatty Liver Disease, Children, Obesity

*Dear Editor,*

In their recent article Kelishadi et al. (1) analyzed in a clear and concise way a number of key aspects of obesity-related NAFLD, deepening in particular the role of gut microflora and its potential therapeutic modulation by probiotics.

From their paper it is apparent that most promises still derive from a relevant number of studies conducted in different NAFLD animal models. When interpreting data obtained in these models one should however remind that other factors like physical activity, social and chemical environment, psychological stress factors and genetics are also important contributors to the development of human NAFLD and to its incredible resistance to treatments as well (2). In this respect Dr. Kelishadi and colleagues' review of four available clinical trials on human population is therefore particularly welcomed. In three studies probiotic treatments appear to improve somehow NAFLD related parameters. The fourth study, published in 2008 by doctor Diehl’s group, instead warned versus probiotics treatment of NAFLD, reporting that steatosis measured by proton magnetic resonance spectroscopy increased in three out of four patients treated with VSL#3 for 4 months (3).

In addition to these meagre evidences, in recent times, Malguarnera et al. showed in histologically monitored NAFLD patients that Bifidobacterium longum + fructo-oligosaccharides (FOS) treatment and lifestyle modification, when compared to lifestyle modification alone, significantly reduced a number of serum inflammatory, metabolic, toxinic and cytolitic parameters, along with improved histological steatosis, and NASH activity index (4). Even more recently, a further open label randomized controlled trial in 20 obese adult patients with biopsy proven NAFLD appeared fairly promising. Patients received a six month formula containing five strains of bacteria (4 lactobacilli + Bifidobacterium bifidum) + FOS. Treatment resulted in reduction of liver fat (P = 0.034) and AST level (absolute change P = 0.021; percentage of baseline value P = 0.008) (5).

In all cases, it should be emphasized that the hitherto available study designs are not homogeneous and data results cannot be easily compared. Study population of two examined series by dr Kelishadi and colleagues for example included different chronic liver diseases in addition to NAFLD (6, 7); probiotics preparations often contained different bacteria strains, and sometimes confounding prebiotics (FOS) and/or vitamins cocktails (4-6); study duration was quite different, and it is still not clear if longer study may be affected by a number of biases like concurrent unpredictable major life style modifications, and at which extent.

Last, but not least, although the Authors reminded the seriousness of epidemiologic data of pediatric NAFLD, they do not refer further to pediatric therapeutic studies targeting gut microbiota. In this regard we think therefore it might be valuable to the discussion completeness also the inclusion of our 2011 study published in Journal of Pediatric Gastroenterology and Nutrition (8). In that double-blind, placebo-controlled pilot study the effects of a short-term (8 weeks) probiotic treatment in 20 children with obesity-related liver disease were evaluated. Patients received Lactobacillus Rhamnosus strain GG (12 billion CFU/day): this probiotic strain was chosen because of the proven safety and efficacy profile in previous pediatric studies. Consistently with available adult data, in our population a multivariate analysis after probiotic treatment revealed a significant decrease in alanine aminotransferase (average variation vs. placebo P = 0.03), with normalization in most (80%) cases, irrespective of changes in BMI z score and visceral fat. We measured anti peptidoglycan-polysaccharide (PGPS) antibodies, an indirect indicator of intestinal bacterial overgrowth. PGPS is a ubiquitous bacterial antigen found in both gram-positive and gram-negative bacteria wall, and anti PGPS production is a sign of bacteria or bacterial membrane translocation through intestine barrier. Interestingly PGPS antibodies were significantly reduced after probiotic treatment (average variation vs. placebo P = 0.03). Serum levels of tumor necrosis factor-alpha, and US bright liver parameters remained stable during the study frame.

Summing up, taking into account also the above additional evidences, we agree with Dr. Kelishadi and colleagues that available animal and human studies altogether may authorize to further support the view of a reasonable therapeutic role of probiotics in adult and also pediatric NAFLD patients not compliant to lifestyle changes, also because of minimal adverse effects and low cost (9). For better clarifying these aspects, future studies still need to be planned on well characterized larger populations, possibly with single probiotic agents, followed by a wash out period, and/or with gut microbiota characterization. The latter, much interestingly, has been defined only very recently both in adult (10)and in pediatric NAFLD (11).

## References

[1] Kelishadi R, Farajian S, Mirlohi M (2013). Probiotics as a novel treatment for non-alcoholic Fatty liver disease; a systematic review on the current evidences.. Hepat Mon..

[2] Vajro P, Lenta S, Pignata C, Salerno M, D’Aniello R, De Micco I (2012). Therapeutic options in pediatric non alcoholic fatty liver disease: current status and future directions.. Italian J Ped..

[3] Solga SF, Buckley G, Clark JM, Horska A, Diehl AM (2008). The effect of a probiotic on hepatic steatosis.. J Clin Gastroenterol..

[4] Malaguarnera M, Vacante M, Antic T, Giordano M, Chisari G, Acquaviva R (2012). Bifidobacterium longum with fructo-oligosaccharides in patients with non alcoholic steatohepatitis.. Dig Dis Sci..

[5] Wong VW, Won GL, Chim AM, Chu WC, Yeung DK, Li KC (2013). Treatment of nonalcoholic steatohepatitis with probiotics. A proof-of-concept study.. Ann Hepatol..

[6] Loguercio C, De Simone T, Federico A, Terracciano F, Tuccillo C, Di Chicco M (2002). Gut-liver axis: a new point of attack to treat chronic liver damage?. Am J Gastroenterol..

[7] Loguercio C, Federico A, Tuccillo C, Terracciano F, D'Auria MV, De Simone C (2005). Beneficial effects of a probiotic VSL#3 on parameters of liver dysfunction in chronic liver diseases.. J Clin Gastroenterol..

[8] Vajro P, Mandato C, Licenziati MR, Franzese A, Vitale DF, Lenta S (2011). Effects of Lactobacillus rhamnosus strain GG in pediatric obesity-related liver disease.. J Pediatr Gastroenterol Nutr..

[9] Vajro P, Paolella G, Fasano A (2013). Microbiota and gut-liver axis: their influences on obesity and obesity-related liver disease.. J Pediatr Gastroenterol Nutr..

[10] Mouzaki M, Comelli EM, Arendt BM, Bonengel J, Fung SK, Fischer SE (2013). Intestinal microbiota in patients with nonalcoholic fatty liver disease.. Hepatology..

[11] Zhu L, Baker SS, Gill C, Liu W, Alkhouri R, Baker RD (2013). Characterization of gut microbiomes in nonalcoholic steatohepatitis (NASH) patients: a connection between endogenous alcohol and NASH.. Hepatology..

